# Impact of School Rurality on Fruit and Vegetable Selection Differs by Sex and Eating Duration for Arizona Schools

**DOI:** 10.1111/jrh.70208

**Published:** 2026-08-20

**Authors:** Mary Curnutte, Alaina L. Pearce, Marc Adams, Traci Grgich, Meg Bruening

**Affiliations:** ^1^ Department of Nutritional Sciences College of Health and Human Development Pennsylvania State University University Park Pennsylvania USA; ^2^ College of Health Solutions Arizona State University Phoenix Arizona USA

**Keywords:** children, fruit and vegetable intake, healthy eating, rurality, school lunch

## Abstract

**Purpose:**

Almost 1 in 5 children live in rural communities, which are more obesogenic than urban communities. Because rural schools have a higher proportion of students participating in the National School Lunch Program compared to urban schools, we aimed to understand rural–urban differences in student fruit and vegetable (FV) selection, consumption, and waste during lunch.

**Methods:**

This secondary analysis used baseline data from a cluster‐randomized trial testing school‐lunch salad bar and FV marketing interventions between 2017 and 2019 across elementary (*n* = 13), middle (*n* = 12), and high schools (*n* = 12) in Arizona. Students’ objective FV selection, consumption, and waste was measured using digital scales to the nearest 2 g. Mixed effects zero‐inflated negative binomial models tested the effect of school rurality (rural vs. non‐rural), adjusted for sociodemographic and within‐school clustering.

**Findings:**

Rural and non‐rural schools did not differ on average FV selection, consumption, or waste. However, sex and eating duration significantly interacted with rurality (*p* < 0.05). In non‐rural schools, girls were 33% more likely to select FVs for lunch compared to boys (OR = 0.43, *z* = –2.73, *p =* 0.009) and slower eaters in non‐rural areas had 1.16 greater odds of selecting FVs compared to faster eaters (OR = 1.16, *z* = –5.65, *p =* 0.002).

**Conclusion:**

Unexpectedly, rurality was not a correlate of students’ FV selection, consumption, or waste during school lunch. However, associations between sex, eating duration, and FV selection in non‐rural schools aligned with the broader scientific literature. These relations were not observed in rural schools, highlighting the need to better understand the influence of sociodemographic factors and eating styles on children's eating behaviors in rural schools.

## 1 Introduction

With 72% of counties in the United States classified as rural [1], nearly 1 in 5 children reside in rural communities [2]. Due to decades of recognition regarding health disparities, such as all‐cause mortality, healthcare accessibility, social services, and food access, between rural and urban youth [2, 3, 4], there is a renewed emphasis on addressing rural health disparities within the National Institutes of Health, as identified by the Institute for Minority Health and Health Disparities’ Strategic Plan for 2021–2025 [5]. This underscores rural areas as a significant source of inequity. Rural youth are more likely to experience persistent poverty, limited access to grocery stores, and limited access to safe spaces for physical activity [6, 7]. Together, these factors contribute to characterizing rural areas as more obesogenic than urban areas [8, 9]. Youth from rural areas are 26% more likely to experience obesity compared to more urban youth [10].

School meals could help address differences between rural and urban youth's diets. School meals provide the highest quality diet among all major food sources in the United States [11] due to the implementation of school nutrition policies such as the Healthy, Hunger‐Free Kids Act [11, 12]. Rural schools are more likely to provide free meals to all children through the Community Eligibility Provision (CEP) of the Healthy, Hunger‐Free Kids Act [13] due to lower socioeconomic status and persistent poverty, which may explain higher participation rates in the National School Lunch Program (NSLP) at rural compared to urban schools [14]. While there is no evidence for differences in the dietary quality of school lunch menus between rural and urban schools [15], as school lunches follow strict federal regulations [16], there are important differences in district‐ or school‐level voluntary nutritional policies in rural compared to urban schools that may influence students’ food choice and intake. Rural areas have fewer policies related to healthy eating outside of the cafeteria and are less likely to ban food marketing or limit portion sizes of high energy‐dense, competitive foods [7, 17]. Students from rural area schools report eating fewer fruits and vegetables (FV) compared to students in suburban and city area schools [18]. When asked about implementation of new or improved nutritional standards, administrators from rural schools report many barriers, including the impact of community/home food environment on FV acceptance among students, food waste, cost, and limited staffing, particularly among school food and nutritional professionals [19, 20, 21]. Additionally, evidence suggests that rural schools often receive less reimbursement from CEP compared to urban schools, resulting in higher per‐pupil cost for school meals, further limiting resources [13]. Together, this suggests that differences in community and school food environments may impact rural school students’ FV intake.

Implementation of NSLP nutrition policies (e.g., Healthy, Hunger‐Free Kids Act) have led to a greater selection of FV at school lunches [22, 23, 24, 25, 26, 27]. However, FV consumption remains below the recommended number of servings [28]. Despite known differences in community and school food environments, it remains unclear how school rurality is related to students’ FV selection, consumption, and waste. Therefore, the present study aims to compare FV selection, consumption, and waste between students attending rural and urban Arizona schools. We hypothesized that lower FV selection and intake would be observed among rural compared to urban school students. As girls report greater liking and consumption of FV compared to boys, possibly due to social and peer influences [29], it was also hypothesized that sex at birth would moderate the effect of rurality on FV selection, consumption, and waste. Lastly, as longer lunch periods are associated with greater selection and consumption of FV [30, 31, 32, 33], we hypothesized that greater time to eat would lead to greater increases in FV selection and consumption among rural compared to urban school students.

## 2 Materials and Methods

This secondary analysis utilizes baseline data from a cluster‐randomized controlled trial examining the efficacy of school‐lunch salad bars and FV marketing conditions to increase students’ FV consumption [34]. The methodology of the cluster RCT study has been previously published [34]. Baseline data collection occurred between fall of 2017 and spring of 2019 in elementary (*n* = 13), middle (*n* = 12), and high schools (*n* = 12) across Arizona. Within each qualifying school (*N* = 37), students were randomly selected to participate in proportion to their representation within a school by grade level. Students were blinded to the purpose of the study and measurement staff were blinded to school intervention status during baseline measures. Students were excluded from the study if they brought lunch from home, were enrolled in special education programs, or were participating in lunch detention. Principals provided written consent prior while acting in *loco parentis* and students gave verbal assent before data collection. This study was approved by the Arizona State University Institutional Review Board.

### 2.1 Protocol

Assented students were provided a pre‐weighed lunch tray with a barcode. As students progressed through the lunch line, their barcode was scanned 3 times: after student assent, after selecting lunch but before eating, and after lunch. Students were asked to eat lunch as usual and were asked not to share food or discard anything. Students returned the tray when they finished eating and were provided a small gift for participating. Research staff measured the weights of individual lunch trays at two points during lunch: once after selecting all lunch items but before eating (i.e., pre‐lunch) and again after eating (i.e., post‐lunch). At both pre‐ and post‐lunch, staff measured individual student trays once with all food items (i.e., entrée, FV, beverage, dessert) and once with FVs only. Measures included tray and FV only weights using digital scales calibrated to 2 g, and pre‐ and post‐lunch photographs to document food items. Tray weights were factored out at pre‐ and post‐lunch to produce net weights. For this study, only FVs that were served cold, such as entrée salads, salsa, hummus, and fresh or canned FVs, were measured. Hot FV items served as part of the entrée and juice were excluded from the operationalization of FVs and were not included in the separate FV tray weights/photographs. Remaining peels and food rinds were included in post‐lunch weights. Any issues with student trays during the eating period (e.g., sharing of food items) or data collection (e.g., spilling of tray items) were recorded and excluded from the sample, as these behaviors can impact the accuracy of the results.

### 2.2 Measures

#### 2.2.1 Sociodemographic Information

Schools and/or districts reported sociodemographic information for students consisting of age, sex, race/ethnicity, free/reduced lunch eligibility, and grade level.

#### 2.2.2 FV Selection, Consumption, and Waste

FV selection was operationalized as the pre‐lunch weight of FV items only. FV consumption was calculated as the difference in post‐ and pre‐FV weights (i.e., excluding other food items). Post‐lunch weights equal to or less than 2 g of pre‐lunch tray weight were treated as zero consumption due to the scale precision range. FV waste was calculated as the percent of selected FV amounts (pre‐lunch FV weight) that remained after the lunch trays were returned (post‐lunch FV tray weight) (i.e., post‐lunch FV weight / pre‐lunch FV weight).

#### 2.2.3 Lunch Environment

Schools provided schedules showing the duration of the lunch period. For each individual student, we calculate the individual time waiting in the lunch line and the total eating duration from the timing of tray barcode scans. Time waiting in the lunch line was determined as the time between the initial assent scan and the pre‐lunch food weight scan. Eating duration was calculated as the difference in time between the pre‐lunch food weight scan and the post‐lunch tray weight scan.

#### 2.2.4 School Rurality

The National Center for Education Statistics (NCES) categorizes schools according to the type of locality in which they are geographically situated: city, suburban, town, and rural. Similar to prior work [20], schools were categorized using the NCES classification as: (1) Urban—schools classified as a city; (2) Suburban—schools classified as suburban; and (3) Rural—schools classified as either towns or rural. Given the low percentage of students from suburban schools (<12%, *n* = 4), primary analyses compared rural schools to non‐rural schools, which included both suburban and urban school students. As such, for this study, we combined suburban and urban schools into non‐rural schools. This approach aligns with the categorization of rural and non‐rural schools from Lin and Fly (2016) [18].

### 2.3 Analytic Approach

Analyses were run R (version 4.1.1) [35] and all analytic scripts are available on Open Science Foundation (osf.io/9kumz/) with data available upon request.

#### 2.3.1 Demographic Characteristics

Differences in sociodemographic and characteristics of interest across rurality (i.e., rural vs. non‐rural) were evaluated using mixed effects one‐way analyses of variance (ANOVAs), adjusting for within‐school clustering for continuous variables (e.g., grade) and cluster‐weight $\chi^{2}$ tests for categorical variables (e.g., sex; estimated using the htestClust package [36]). Mixed‐effects models adjusted for within‐school clustering examined differences in lunch environment characteristics (e.g., lunch duration) by rurality.

#### 2.3.2 FV Selection, Consumption, and Waste

Given the high proportion of zero values for FV selection, consumption, and waste, mixed effects zero‐inflated negative binomial models adjusting for student observations clustered within schools were used to test associations with school rurality (estimated using the GLMMadaptive package [37]). Because the zero portion of the consumption model represents students who wasted 100% of their FVs (i.e., 0 g consumed), the FV waste (count portion) model only includes students who did not waste 100% of their FVs. All models were adjusted for school level (i.e., elementary, middle, high), race/ethnicity, free/reduced price lunch, and lunch period duration, as covariates of no interest for both the count and zero‐inflated portions of the models.

#### 2.3.3 Moderation Analyses

As both sex [29] and eating duration [38] are known to impact FV intake at school lunch, sex and eating duration were interacted to be tested as moderators of the effect of rurality for FV selection, consumption, and waste. As with the overall model, all moderation analyses used mixed‐effects zero‐inflated negative binomial models clustered within schools and included school‐level, race/ethnicity, free/reduced‐price lunch, and lunch duration as covariates of no interest. Eating duration was centered at the mean for moderation analyses.

## 3 Results

### 3.1 Demographic Characteristics

Distributions of sex, race/ethnicity, and qualification of free/reduced lunch did not differ significantly by rurality (*p* > 0.16) (Table 1). There were also no statistically significant differences by rurality in school lunch duration (*F*(1, 35) = 1.48, *p =* 0.232) or time spent waiting in line (*F*(1, 35) = 0.022, *p =* 0.882) (Table 1).

**TABLE 1:** Demographic characteristics and key study outcomes.

	Overall *N* = 2832 (100%)	Rural *N* = 740 (26.1%)	Non‐rural *N* = 2092 (73.9%)
	*N* (%)	*N* (%)	*N* (%)
Sex			
Female	1328 (46%)	356 (48%)	972 (46%)
Male	1504 (53%)	484 (52%)	1120 (54%)
School level			
Elementary	1006 (36%)	224 (30%)	782 (37%)
Middle school	818 (29%)	238 (32%)	580 (28%)
High school	1008 (36%)	278 (38%)	730 (35%)
Race/ethnicity			
Hispanic/Latinx	1751 (62%)	430 (58%)	1321 (63%)
White	713 (25%)	201 (27%)	512 (24%)
Black/African American	160 (6%)	38 (5%)	122 (6%)
Other	208 (7%)	71 (10%)	137 (7%)
Lunch payment			
Free/reduced	2273 (80%)	651 (88%)	1622 (78%)
Paid	559 (20%)	89 (12%)	470 (22%)

	Mean (SD)	Mean (SD)	Mean (SD)
Grade	6.9 (3.3)	7.1 (3.4)	6.8 (3.2)
Lunch period, min	27.8 (9.5)	25.0 (6.5)	26.5 (7.1)
Eating duration, min	10.6 (4.1)	10.3 (4.1)	9.9 (3.3)
Time in lunch line, min	5.4 (4.4)	5.5 (2.6)	6.0 (10.1)
Unknown, *N* (%)	83 (3%)	46 (6%)	0 (0%)
F/V selected, *N* (%)			
None	399 (14%)	73 (10%)	106 (32%)
Some	2433 (86%)	667 (90%)	229 (68%)
F/V consumed,^a^ *N* (%)			
None	309 (13%)	95 (14%)	31 (14%)
Any	2124 (87%)	572 (86%)	198 (86%)
Selected,^a^ g	135.8 (68.9)	141.4 (55.8)	122.5 (62.9)
Consumed,^a^ g	58.1 (54.9)	66.8 (56.4)	46.6 (49.3)
Waste,^a^ g	77.7 (65.7)	74.6 (59.6)	75.9 (57.3)
Waste,^a^ %	55.8 (35.3)	53.3 (35.7)	60.0 (34.6)

*Note*: Percentages may not sum to 100% due to rounding error.

Abbreviation: SD, standard deviation.

^a^Sample restricted to those that selected any fruit/vegetables.

### 3.2 Effect of School Rurality on FV Selection, Consumption, and Waste

#### 3.2.1 Effect on Consuming Zero Grams of FV or Some FV

No differences were observed between rural and non‐rural students for the likelihood of selecting, consuming FVs, or wasting selected FV (Table 2). There was a difference by school level for the likelihood of selecting FVs, such that middle and high schoolers were less likely to select FV compared to elementary schoolers (*p*s < 0.001). All but 5 students in elementary school selected a FV. Compared to students who identified as Hispanic/Latinx (the referent group as 62% of the sample identified as Hispanic/Latinx), students identifying as White were less likely to select FVs (OR = 0.64, *p =* 0.015). Lastly, those who paid full price for school lunch were less likely to select FVs compared to students whose families qualified for free/reduced lunch (OR = 0.63, *p =* 0.03).

**TABLE 2:** Relationship of rurality on fruit and vegetable selection, consumption, and waste among school students in Arizona (*n* = 2832).

	Count model								
	Selection			Consumption			Percent waste		
	β (SE)	*p*	IRR	β (SE)	*p*	IRR	β (SE)	*p*	IRR
School level (ref = Elementary)									
Middle school	−0.14 (0.13)	0.276	0.87	−**0.38 (0.13)**	**0.003**	**0.68**	**0.12 (0.11)**	**0.035**	**1.27**
High school	−0.13 (0.13)	0.338	0.88	−**0.49 (0.13)**	**<0.001**	**0.61**	**0.31 (0.12)**	**0.008**	**1.35**
Race/ethnicity (ref = Hispanic/Latinx)									
White	−0.02 (0.03)	0.455	0.98	−0.06 (0.06)	0.317	0.94	0.01 (0.05)	0.874	1.01
Black	0.03 (0.04)	0.341	1.03	−0.11 (0.09)	0.253	0.90	0.11 (0.07)	0.113	1.11
Other	−0.04 (0.03)	0.195	0.96	−**0.21 (0.08)**	**0.011**	**0.81**	**0.16 (0.06)**	**0.014**	**1.18**
Paid lunch status (ref = free/reduced)	0.05 (0.03)	0.101	1.05	0.07 (0.07)	0.302	1.08	0.01 (0.05)	0.892	1.01
Lunch duration	0.01 (0.01)	0.184	1.00	0.01 (0.01)	0.467	1.00	0.01 (0.01)	0.689	1.00
Rurality (ref = rural)	0.09 (0.13)	0.452	1.10	0.15 (0.12)	0.225	1.16	−0.04 (0.11)	0.740	0.96

	Zero model								
	β (SE)	*p*	OR	β (SE)	*p*	OR	β (SE)	*p*	OR
School level (ref = Elementary)									
Middle school	−**4.20 (1.21)**	**<0.001**	**0.01**	−0.25 (0.30)	0.414	0.78	−0.30 (0.62)	0.627	0.74
High school	−**5.11 (1.24)**	**<0.001**	**0.01**	−0.41 (0.32)	0.196	0.66	−1018 (0.64)	0.064	0.31
Race/ethnicity (ref = Hispanic/Latinx)									
White	−**0.45 (0.19)**	**0.015**	**0.64**	−0.12 (0.19)	0.521	0.88	0.14 (0.29)	0.634	1.15
Black	−0.41 (0.33)	0.216	0.66	−0.42 (0.27)	0.129	0.66	−0.66 (0.35)	0.060	0.52
Other	0.02 (0.29)	0.939	1.02	−0.25 (0.25)	0.319	0.78	−**0.92 (0.29)**	**0.001**	**0.40**
Paid lunch status (ref = free/reduced)	−**0.46 (0.21)**	**0.029**	**0.63**	−0.11 (0.22)	0.609	0.89	−0.23 (0.30)	0.433	0.79
Lunch duration	−0.02 (0.01)	0.283	0.98	−0.01 (0.01)	0.896	1.00	−0.02 (0.02)	0.292	0.98
Rurality (ref = rural)	0.06 (0.95)	0.996	1.06	0.18 (0.28)	0.520	1.19	−0.32 (0.58)	0.583	0.72

*Note*: Bold text indicates significance of *p* < 0.05.

Abbreviations: IRR, incidence rate ratio; OR, odds ratio.

#### 3.2.2 Effect on Amount of FV Selected, Consumed, or Wasted Among Those Who Selected, Consumed, or Wasted Any FVs

Among those who selected, consumed, or wasted any FVs, there was no overall effect of rurality on the *amounts* of FV selected, consumed, and percent wasted. However, among students who consumed any FVs, the amounts consumed and percent wasted differed by school level and race/ethnicity (Table 2). Middle and high schoolers who consumed any FVs, consumed, on average, 30 g and 43 g more FVs compared to elementary schoolers (*p*s < 0.003), with no difference by rurality. Students who identified as a race other than White or Black consumed 15 g more FVs compared to those who identified as Hispanic/Latinx (*p =* 0.011); there was no difference in FV consumption between students identifying as Hispanic/Latinx and Black and White students (*p*s > 0.244). Similar to FV consumption, among those who wasted anything, the percent waste was 11 and 15 percentage points lower for middle and high school students compared to elementary school students (*p*s < 0.039) and 8 points lower for students who identify as a race other than White or Black compared to those who identified as Hispanic/Latinx (*p =* 0.11). Therefore, although rurality was not associated with FV selection, consumption, or waste overall, there were differences by school level, race/ethnicity, and free/reduced lunch status.

### 3.3 Moderation of Rurality by Sex

Sex moderated the effect of rurality on the likelihood of selecting any FVs (Table 3). There was no difference between male and female students in rural schools (OR = 1.47, *z* = 1.39, *p =* 0.165). However, in non‐rural schools, females were 33% more likely than males to select some FVs (OR = 0.43, *z* = –2.73, *p =* 0.009). Sex did not moderate the effect of rurality on the *amount* of FVs selected in the count model.

**TABLE 3:** Moderation of rurality by sex for fruit and vegetable selection, consumption, and waste among Arizona students (*n* = 2832).

	Count model								
	Selection			Consumption			Percent waste		
	β (SE)	*p*	IRR	β (SE)	*p*	IRR	β (SE)	*p*	IRR
School level (ref = Elementary)									
Middle school	−0.14 (0.13)	0.296	0.87	−**0.38 (0.13)**	**0.003**	**0.68**	**0.23 (0.11)**	**0.039**	**1.27**
High school	−0.12 (0.13)	0.359	0.88	−**0.50 (0.14)**	**<0.001**	**0.61**	**0.31 (0.12)**	**0.007**	**1.37**
Race/ethnicity (ref = Hispanic/Latinx)									
White	−0.02 (0.03)	0.402	0.98	−0.06 (0.06)	0.352	0.94	0.01 (0.05)	0.829	1.01
Black	0.04 (0.04)	0.272	1.04	−0.11 (0.09)	0.244	0.90	0.11 (0.07)	0.114	1.11
Other	−0.04 (0.03)	0.198	0.96	−**0.21 (0.08)**	**0.011**	**0.81**	**0.16 (0.06)**	**0.011**	**1.18**
Lunch payment (ref = free/reduced)	0.05 (0.03)	0.096	1.05	0.07 (0.07)	0.284	1.08	−0.01 (0.05)	0.962	1.00
Lunch duration	0.01 (0.01)	0.220	1.00	0.01 (0.01)	0.415	1.00	0.01 (0.01)	0.863	1.00
Rurality (ref = rural)	0.08 (0.13)	0.542	1.08	0.12 (0.13)	0.379	1.12	0.05 (0.11)	0.665	1.05
Sex (ref = F)	0.01 (0.03)	0.934	1.00	−0.11 (0.08)	0.183	0.90	**0.19 (0.06)**	**0.001**	**1.20**
Rurality × sex (ref = non‐rural × F)	0.03 (0.04)	0.386	1.03	0.06 (0.09)	0.511	1.06	−**0.16 (0.07)**	**0.017**	**0.85**

	Zero model								
	β (SE)	*p*	OR	β (SE)	*p*	OR	β (SE)	*p*	OR
School level (ref = Elementary)									
Middle school	−**4.23 (1.21)**	**<0.001**	**0.01**	−0.25 (0.30)	0.400	0.78	−0.32 (0.62)	0.609	0.73
High school	−**5.12 (1.24)**	**<0.001**	**0.01**	−0.42 (0.32)	0.192	0.66	−1.22 (0.64)	0.054	0.29
Race/ethnicity (ref = Hispanic/Latinx)									
White	−**0.45 (0.19)**	**0.016**	**0.64**	−0.12 (0.19)	0.538	0.88	0.17 (0.29)	0.554	1.19
Black	−0.41 (0.33)	0.224	0.67	−0.43 (0.27)	0.121	0.65	−0.68 (0.29)	0.056	0.51
Other	0.02 (0.29)	0.947	1.02	−0.24 (0.25)	0.342	0.79	−**0.89 (0.29)**	**0.002**	**0.41**
Lunch payment (ref = free/reduced)	−**0.46 (0.21)**	**0.030**	**0.63**	−0.11 (0.22)	0.625	0.89	−0.22 (0.30)	0.454	0.80
Lunch duration	−0.02 (0.01)	0.276	0.98	−0.01 (0.01)	0.921	1.00	−0.02 (0.02)	0.387	0.98
Rurality (ref = rural)	0.52 (0.96)	0.592	1.67	0.30 (0.32)	0.339	1.35	−0.37 (0.64)	0.561	0.69
Sex (ref = F)	0.38 (0.76)	0.165	1.47	0.07 (0.24)	0.339	1.08	−0.46 (0.37)	0.215	0.63
Rurality × sex (ref = non‐rural × F)	−**0.83 (0.32)**	**0.009**	**0.43**	−0.24 (0.29)	0.406	0.79	0.05 (0.42)	0.915	1.04

*Note*: Bold text indicates significance of *p* < 0.05.

Abbreviations: IRR, incidence rate ratio; OR, odds ratio.

Sex did not moderate the effect of rurality on consumption, but we observed a significant sex by rurality interaction for the percent FVs wasted in the count model (IRR = 0.85, *p* = 0.016, *p* = 0.017). There was no difference in the percent of FV wasted by sex for non‐rural schools (*p* = 0.215); however, in rural schools, the percent waste was 9 points greater among females compared to males (IRR = 1.20, *z* = 3.27, *p =* 0.001). Taken together, this suggests that while there was no difference in the selection of FVs by sex in rural schools, females wasted a greater proportion of selected FVs compared to males in rural schools.

### 3.4 Moderation of Rurality by Eating Duration

Eating duration significantly moderated the effect of rurality on the likelihood of selecting any FVs, but it did not moderate the effect of rurality on the amount of overall grams of FVs consumed (Table 4). While eating duration was not associated with odds of selecting any FVs among rural school students (OR = 0.97, *z* = 0.76, *p =* 0.563), longer eating duration was associated with higher odds of selecting any FVs in non‐rural schools (OR = 1.16, *z* = –5.65, *p =* 0.002). In non‐rural schools, each additional minute of eating duration increased the odds of consuming some FVs by 12%. While there was no interaction between eating duration and rurality for FV consumption, eating for longer was associated with higher odds of consuming any along with higher amounts of FVs in rural schools (OR = 1.11, *z* = –2.42, *p* = 0.016; IRR = 0.96, *z* = 3.20, *p* = 0.001). There was a significant eating duration by rurality interaction for percent waste such that longer eating duration was associated with less waste at rural but not at non‐rural schools (Table 4). In rural schools, each minute longer spent eating was associated with a 2‐point decrease in percent waste (IRR = 0.96, *z* = –4.92, *p* < 0.001), corresponding to a 1.5 g decrease in FV wasted.

**TABLE 4:** Moderation of rurality by eating duration for fruit and vegetable selection, consumption, and waste.

	Count model								
	Selection			Consumption			Percent waste		
	β (SE)	*p*	IRR	β (SE)	*p*	IRR	β (SE)	*p*	IRR
School level (ref = Elementary)									
Middle school	−0.14 (0.15)	0.311	0.87	−**0.44 (0.14)**	**0.002**	**0.65**	**0.28 (0.10)**	**0.006**	**1.32**
High school	−0.10 (0.14)	0.452	0.90	−**0.49 (0.15)**	**0.001**	**0.61**	**0.29 (0.11)**	**0.006**	**1.33**
Race/ethnicity (ref = Hispanic/Latinx)									
White	−0.01 (0.03)	0.622	0.99	−0.04 (0.07)	0.606	0.96	−0.03 (0.05)	0.541	0.97
Black	0.03 (0.04)	0.485	1.03	−0.14 (0.10)	0.151	0.87	0.12 (0.07)	0.088	1.14
Other	−0.03 (0.03)	0.413	0.97	−**0.23 (0.09)**	**0.007**	**0.79**	**0.18 (0.07)**	**0.005**	**1.14**
Lunch payment (ref = free/reduced)	0.05 (0.03)	0.080	1.05	0.08 (0.07)	0.305	1.08	0.01 (0.05)	0.805	1.01
Lunch duration	0.01 (0.01)	0.463	1.00	0.01 (0.01)	0.721	1.00	0.01 (0.003)	0.090	1.01
Rurality (ref = rural)	0.09 (0.13)	0.527	1.09	0.14 (0.13)	0.295	1.15	−0.06 (0.10)	0.513	0.94
Eating duration^a^	0.01 (0.01)	0.923	1.00	−**0.04 (0.01)**	**0.001**	**0.96**	**0.04 (0.01)**	**<0.001**	**1.04**
Rurality (ref = non‐rural) × Eating duration^a^	−0.01 (0.01)	0.424	1.00	0.02 (0.02)	0.235	1.02	−**0.04 (0.01)**	**<0.001**	**0.96**

	Zero model								
	β (SE)	*p*	OR	β (SE)	*p*	OR	β (SE)	*p*	OR
School level (ref = Elementary)									
Middle school	−**3.83 (1.27)**	**0.002**	**0.02**	−0.19 (0.35)	0.584	0.83	−0.27 (0.66)	0.684	0.76
High school	−**4.90 (1.30)**	**<0.001**	**0.01**	−0.54 (0.37)	0.144	0.58	‐.098 (0.69)	0.157	0.38
Race/ethnicity (ref = Hispanic/Latinx)									
White	−**0.42 (0.21)**	**0.042**	**0.66**	−0.21 (0.23)	0.368	0.81	0.16 (0.31)	0.612	1.18
Black	−0.67 (0.36)	0.070	0.52	−0.35 (0.32)	0.276	0.70	−0.58 (0.37)	0.114	0.56
Other	0.19 (0.32)	0.556	1.20	−0.40 (0.27)	0.141	0.67	−**0.93 (0.30)**	**0.002**	**0.40**
Lunch payment (ref = free/reduced)	−**0.57 (0.24)**	**0.016**	**0.56**	−0.11 (0.26)	0.678	0.90	−0.26 (0.31)	0.392	0.77
Lunch duration	−0.02 (0.01)	0.152	0.98	−0.01 (0.01)	0.782	1.00	−0.03 (0.02)	0.151	0.97
Rurality (ref = rural)	0.28 (1.01)	0.781	1.32	0.06 (0.33)	0.847	1.06	−0.31 (0.62)	0.619	0.74
Eating duration^a^	−0.02 (0.04)	0.563	0.97	**0.11 (0.04)**	**0.016**	**1.11**	−0.04 (0.06)	0.437	0.96
Rurality (ref = non‐rural) × Eating duration^a^	**0.15 (0.05)**	**0.002**	**1.16**	−0.01 (0.05)	0.876	0.99	0.02 (0.06)	0.715	1.02

*Note*: Bold text indicates significance of *p* < 0.05.

Abbreviations: IRR, incidence rate ratio; OR, odds ratio.

^a^Eating duration was centered and the grand mean.

## 4 Discussion

Previous studies have examined the impact of school rurality on the nutritional content of school menus [15], use of salad bars [20], and self‐reported FV intake [18]. The present study examined rurality as a correlate of objectively measured FV selection, consumption, and waste for individual students. In contrast to our hypothesis, no general effect of school rurality on objectively measured FV selection, consumption, or waste was observed. However, in line with our hypotheses, the effect of rurality was moderated by both sex and eating duration. While girls were more likely to select any FV compared to males in more non‐rural schools, there was no sex difference in selection for rural schools. While there was no effect of rurality on *amount* of FV consumed, eating for longer was associated with greater odds of consuming any FV for rural but not non‐rural schools. While girls were more likely to waste FV in rural than in non‐rural schools, longer eating durations were associated with less waste in rural than in non‐rural schools. Together, this suggests that sex and eating duration play differing roles in their influence on FV waste in rural versus non‐rural schools.

Sex was a significant moderator of the effect of school rurality on the selection of some FVs. Our previous research has demonstrated that there is higher likelihood of consuming FVs if they are selected and on the tray [34, 39]. Girls of all ages are more likely to try, consume, and like FVs compared to boys [29, 40, 41]. As girls are more responsive to parental and peer modeling of healthy behaviors [29, 42], one explanation of the lack of sex differences in FV selection among rural students is more limited modeling of FV intake due to barriers in rural community and home food environments [43, 44]. Rural adults report lower FV intake [43, 44]. Lower consumption of FV intake in rural communities is related to distance from grocery stores and supermarkets, perception that FVs cost too much and spoil too quickly, and limited preferences for FVs [17, 43, 44, 45, 46]. Another possible explanation is that rural and non‐rural schools differ in the implementation of voluntary healthy eating initiatives and policies [7, 17]. School and district‐level policies supportive of healthy eating have been shown to be less robust in rural areas and the nutritional quality of foods served in schools outside of the NSLP has been shown to be lower in rural areas [7, 17]. Differences could be seen in schools requiring students to take multiple food groups to create a “complete” meal for reimbursement or in the preparation and variety of FVs. This difference could be due to fewer resources in rural schools as a result of their lower enrollments [13, 47], the less availability of healthy foods in rural areas which may impact student preferences in schools [46], or the higher cost of high‐quality FVs [16]. Cultural norms in rural areas may be less supportive of healthy eating leading to lower intrinsic motivation to implement such policies [7]. Gendered perceptions and motivations for healthy eating begin to emerge in childhood such that girls focus more on appearance and weight status [48] and perceive their peers to eat more FV compared to boys [49]. Greater emphasis on healthy eating in non‐rural schools may lead to more gendered perceptions of peer behavior and increase social influence, which may contribute to girls being more likely than boys to select FVs. However, more research is needed to better understand how rural–non‐rural differences in community, school, and home food environments differentially impact eating behaviors for boys and girls.

Similar to sex, individual differences in eating duration had a differing role on the likelihood of selecting no FVs for rural versus non‐rural schools. Students who spent more time eating were more likely to select any FVs in non‐rural schools. This finding aligns with data showing that longer eating and lunch duration are associated with greater FV selection and consumption [30, 31, 32]. As longer eating duration was associated with greater consumption of any FVs for rural students in this study, it is possible that differences in FV selection were due to cultural differences in the acceptance of FVs between more rural and non‐rural communities. While the likelihood of selecting FVs was not impacted by eating duration in rural schools, among those who selected FVs, longer eating duration was associated with less FV waste for rural but not non‐rural students. Therefore, while eating duration had a greater association on the likelihood of selecting some of the FVs for students at non‐rural schools, it had a greater influence likelihood of consuming any FV and reducing FV waste in more rural schools. Together, these findings suggest that school nutrition programs around student eating duration need to be tailored differently to motivate FV selection in rural and non‐rural schools. The absence of resources for nutrition programming in rural schools [19, 20, 21] and if there are differing lunch period lengths between the settings, which has not yet been studied, may influence FV eating behavior in rural versus non‐rural schools. As financial constraint has been identified as a challenge in rural school meals [19, 21], increasing lunch duration could be a cost‐effective intervention to impact the likelihood of any FV consumption. More work is needed to better understand differences in rural and non‐rural students’ motivations and perceptions of school lunch to improve FV selection and intake.

Several methodological considerations should be considered. This study is unique in its use of objectively measured weights of FV selected, consumed, and wasted in both rural and non‐rural schools in Arizona. The sample was relatively large and demographically diverse, providing insights on unstudied groups. However, there are also limitations that should be considered when interpreting or generalizing these results. This study was a secondary analysis and, therefore, opportunistic and relied on the existing design and methods of the larger trial. Baseline measures were conducted once, which may not reflect usual intake; however, efforts were made to ensure as much similarity in menu items across schools as possible. As a secondary data analysis, the number of rural (*n* = 740) and non‐rural schools (*n* = 2092) was not balanced, but given the large sample size, this may not have significantly affected the results. The operationalization of FVs in this study also only considered cold fresh or side FV options, excluding FVs integrated into entrees or included as warm side (e.g., green beans). There is a need for more research that is a priori focused and powered to investigate rural–non‐rural differences in school food environments and healthy eating. Additionally, to accurately measure FV, students were told not to share food. However, sharing food is a normal part of student lunch. Future research could investigate sharing and social influence on FV consumption. While we see a rural–non‐rural difference in eating patterns, qualitative studies can explore what mechanisms may drive this difference. More research is needed to determine if and how lunch periods differ between rural and non‐rural schools to better understand the differences in eating duration as reported in the current study. Understanding the drivers of rural–non‐rural differences can inform interventions to equitably promote FV consumption across all geographic settings.

## 5 Conclusion

While rural and non‐rural schools did not differ on average FV selection, consumption, or waste, this study found important rural–non‐rural differences in the roles of sex and eating duration for FV selection. In non‐rural schools, girls and students who spent longer eating were more likely to select FVs for lunch compared to boys and those who spent less time eating, respectively. Both these findings align with the larger literature. Girls report greater liking and consumption of FVs compared to boys [29, 40, 41]. Additionally, both longer eating duration and lunch duration have been associated with greater FV selection and consumption at school lunches [30, 31, 32]. These lacks of observed associations by rurality highlight the need for more research to better understand the influence of sociodemographic factors and eating styles on children's eating behaviors in rural schools. Given that almost two‐thirds of rural communities are designated as primary care Health Professional Shortage Areas [4], schools in rural communities represent important community centers for the addressing health disparities. More research is needed to better tailor healthy eating and nutritional education intervention programs to rural schools and communities.

## Funding

This work was supported by the National Institutes of Health [R01HL139120; MPIs Bruening/Adams]. The funding agency had no involvement in any aspect of this study or manuscript.

## Ethics Statement

This study was approved by the Arizona State University (ASU) Institutional Review Board (study protocol [STUDY0000582]).

## Conflicts of Interest

Since completing the data collection for this manuscript, Bruening and Adams have received funding from the National Dairy Council to examine how milk selection is related to fruit and vegetable consumption and waste.
